# Lytic bacteriophage have diverse indirect effects in a synthetic cross-feeding community

**DOI:** 10.1038/s41396-019-0511-z

**Published:** 2019-10-02

**Authors:** Lisa Fazzino, Jeremy Anisman, Jeremy M. Chacón, Richard H. Heineman, William R. Harcombe

**Affiliations:** 1 Department of Microbiology and Immunology, University of Minnesota, Minneapolis, MN USA; 2 BioTechnology Institute, University of Minnesota, Minneapolis, MN USA; 3 College of Continuing and Professional Studies, University of Minnesota, Minneapolis, MN USA; 4 Ecology, Evolution, and Behavior, University of Minnesota, Minneapolis, MN USA; 5 Biology Department, Kutztown University, Kutztown, PA USA

**Keywords:** Microbial ecology, Microbial ecology, Bacteriophages

## Abstract

Bacteriophage shape the composition and function of microbial communities. Yet it remains difficult to predict the effect of phage on microbial interactions. Specifically, little is known about how phage influence mutualisms in networks of cross-feeding bacteria. We mathematically modeled the impacts of phage in a synthetic microbial community in which *Escherichia coli* and *Salmonella enterica* exchange essential metabolites. In this model, independent phage attack of either species was sufficient to temporarily inhibit both members of the mutualism; however, the evolution of phage resistance facilitated yields similar to those observed in the absence of phage. In laboratory experiments, attack of *S. enterica* with P22*vir* phage followed these modeling expectations of delayed community growth with little change in the final yield of bacteria. In contrast, when *E. coli* was attacked with T7 phage, *S. enterica*, the nonhost species, reached higher yields compared with no-phage controls. T7 infection increased nonhost yield by releasing consumable cell debris, and by driving evolution of partially resistant *E. coli* that secreted more carbon. Our results demonstrate that phage can have extensive indirect effects in microbial communities, that the nature of these indirect effects depends on metabolic and evolutionary mechanisms, and that knowing the degree of evolved resistance leads to qualitatively different predictions of bacterial community dynamics in response to phage attack.

## Introduction

Bacteriophage significantly influence microbial community structure and function [1]. Phage limit the size of bacterial populations, which can change microbial community composition. For example, phage kill >20% of marine bacteria every day [2]. Viral infection of bacterial populations not only impacts the composition of bacterial communities, but also influences emergent community functions such as the rate at which nutrients are converted into biomass [3]. As a result, phage critically influence biogeochemical cycling [4], biotechnology [5], the food industry [6], and human health [7, 8]. Despite the importance of phage in microbial communities, we cannot reliably predict the impact of phage on the composition or function of communities. As we strive to manage microbial communities, we must improve our understanding of responses to phage infection in multi-species systems.

Phage alter competitive bacterial communities by changing the species abundance. When phage kill dominant competitors, weaker competitors that are resistant to phage can flourish, changing species ratios, which can change community function [9–12]. Sometimes, species ratios rapidly revert to pre-phage frequencies once a host evolves phage resistance, but costs of resistance can generate persistent changes in competitive community composition following phage addition [9]. However, phage attack of one species often has little impact on total community biomass. In communities of competitors, a reduction in host biomass is typically compensated for by the growth of nonhost competitors [13]. Taken together, in competitive systems, phage alter species ratios, but have little impact on total microbial biomass.

Much less is understood about how phage influence cooperative networks in microbial communities. Microbial communities are often organized into cross-feeding webs in which each species relies on metabolites excreted by others [14, 15]. Networks of metabolic dependencies have been described in marine, terrestrial, and human-associated microbial communities [14]. While phage are likely present in all of these systems, the impact of phage on the composition and function of cross-feeding microbial communities remains under-studied.

However, the response of cross-feeding communities to abiotic disturbances may inform how cross-feeding communities respond to phage infection. In the absence of disturbance, obligate mutualism typically constrains species ratios such that communities converge on an equilibrium ratio from any initial ratio [16, 17]. This constraint on final species ratios means that limiting one species should indirectly inhibit cross-feeding partners, thereby decreasing total community biomass, but maintaining species ratios [18]. For example, antibiotic treatment of a three-species cross-feeding community that inhibited the most sensitive member inhibited growth of all members of the community by depriving community partners of cross-fed nutrients [19]. Therefore, our null hypothesis is that phage infection on one member of a cooperative network will limit growth of the entire cross-feeding network but will not change species ratios.

Yet, the null hypothesis that phage infection will alter cooperative community biomass but not composition has several underlying assumptions that may not hold. First, it assumes that bacteria obtain nutrients directly from the secretions of bacterial partners. Yet there is a rich body of literature suggesting that phage-mediated cell lysis releases nutrients into the environment. Indeed, this ‘viral shunt’ is thought to play a major role in global nutrient cycling [20–22] and may alter species interactions [23, 24]. Second, the hypothesis overlooks possible ecological consequences of the evolution of phage resistance. For example, it assumes that phage resistance does not alter the exchange of cross-fed nutrients. If phage resistance causes changes in either nutrient secretion or uptake, it could alter species ratios, potentially changing community function.

**Fig. 1:**
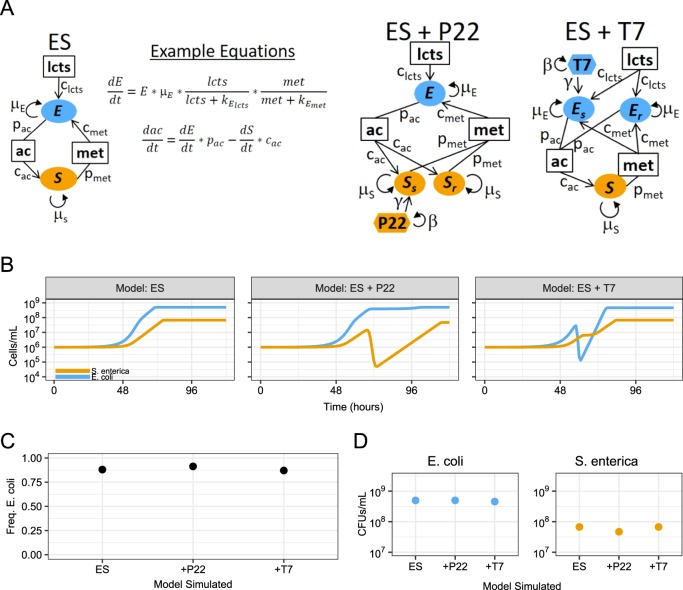
Mathematical model predicting consequences of phage infection of cooperative co-cultures. **a** Schematic of models for systems with *E. coli* (E), *S. enterica* (S), and phage (T7 or P22). Bacteria are represented by ovals. Phage sensitivity (s) or resistance (r) is indicated by subscripts. Phage are indicated by hexagons and are colored to match their bacterial host. Boxes indicate metabolites (lcts lactose, ac acetate, met methionine). Parameters are next to interaction arrows. c_x_ =  consumption rate of subscript nutrient, p_x_ = production rate of subscript nutrient, μ_x_ =  growth rate (h^−1^), β =  burst size, γ =  adsorption constant. **b** Simulated growth curves with and without phage treatment. Yellow = *S. enterica*, blue = *E. coli*. **c** Species ratios represented with frequency of *E. coli* at time = 125 h. **d** Final densities of bacteria (cells/ml) in mathematical models at time = 125 h

In this study, we sought to determine the effects of phage attack on cooperative communities by combining resource-explicit mathematical modeling and wet-lab experiments of a synthetic cross-feeding co-culture of *Escherichia coli* and *Salmonella enterica* [17, 25]. An *E. coli* strain auxotrophic for methionine was paired with a *S. enterica* strain that was evolved to secrete methionine [25]. The pair forms an obligate mutualism in lactose minimal medium, as *S. enterica* cannot consume lactose and instead relies on acetate excreted by *E. coli* during overflow metabolism. Grown under these conditions, these bacteria are a simple two-species cooperative community. To this community, we added either *an E. coli*-specific (T7) or *S. enterica*-specific (P22*vir*) lytic phage and tracked community responses (Fig. 1a). As a null hypothesis, we predicted that cross-feeding would constrain species ratios and therefore targeted phage attack would inhibit growth of the entire community. However, we anticipated that phage resistance would evolve, making biomass reduction temporary. We found that both phage delayed community growth, but neither phage reduced final host yields and T7 infection of *E. coli* led to surprising changes in species ratios.

## Results

### Resource-explicit model suggests phage have little impact on final community composition

We used a resource-explicit model to predict how a cooperatively growing bipartite bacterial community responds to lytic phage attack during batch growth. We modeled densities of *E. coli* (E), *S. enterica* (S), and phage (T7 or P22*vir*), as well as cross-fed metabolite concentrations (Fig. 1a and Supplementary Methods). Growth of each bacterial species was a function of maximum growth rate (μ_x_) and Michaelis–Menten saturation parameters (k_m_) of essential metabolites. Bacterial death due to phage infection was modeled as a linear interaction between phage and host, and modified by an adsorption (i.e., predation) constant (γ). Phage attack generated new phage particles at a rate set by the burst size (β). Phage-resistant hosts (E_R_ or S_R_) were initiated at a frequency of 0.1% in each bacterial population so that resistance alleles increased in frequency during phage infection. The cost of resistance, if any, was represented by a smaller growth rate (μ_x_) of resistant bacteria. Parameters were informed by the literature and adjusted to match experimental observations in the absence of phage (Supplementary Table 1).

When modeled in the absence of phage, the community converged to 88% *E. coli* regardless of the starting bacterial ratio, consistent with previous wet-lab observations (Fig. 1c and Supplementary Fig. 1) [17]. In the absence of phage, sensitive and resistant bacterial genotypes increased in density with relative frequencies determined by the cost of resistance (Supplementary Fig. 2).

The model predicted that the presence of phage increases the time required for the community to reach carrying capacity, but has little impact on the final species yields. Both T7 and P22*vir* rapidly killed all sensitive hosts (Fig. 1b and Supplementary Fig. 2). The reduction in the host population reduced the amount of cross-feeding, thereby temporarily stalling community growth. However, phage-resistant hosts rapidly increased in abundance, allowing the community to reach carrying capacity. Host species reached 91% to 70% of the final densities as a result of phage attack (Fig. 1d). This reduction is because sensitive host cells consume resources before they are killed by phage, and fewer resources are therefore available for growth of the resistant host. However, sensitive hosts are killed before they consume many resources. No change was observed in the final abundance of the nonhost bacteria. Furthermore, reducing maximum growth rates of phage-resistance genotypes as a proxy for the cost of resistance did not change yields, but did cause small delays of community growth (Supplementary Fig. 2a, b).

### In wet-lab experiments, T7 infection changed species ratios while P22*vir* infection did not

Using our wet-lab experimental cross-feeding system, we tested the mathematical model prediction that phage attack would delay growth but have little impact on the final community. Communities were started with a multiplicity of infection (MOI) of ~0.01 (Supplementary Table 2). Any resistant host cells arose via mutation during cooperative community growth; they were not intentionally seeded into the host population. Growth of each bacterial species was tracked with a unique fluorescent marker which could be converted to a species-specific OD (Supplementary Fig. 3) [19]. After growth, co-cultures were plated for *E. coli* and *S. enterica* colony-forming units (CFUs), and T7 or P22*vir* plaque-forming units (PFUs).

**Fig. 2:**
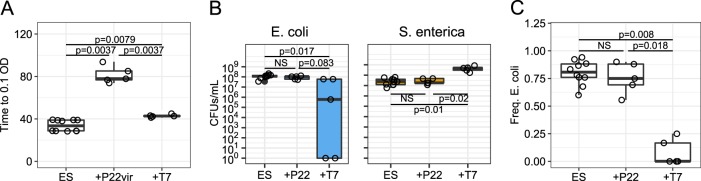
Experimental data of cooperative co-culture growth and the effect of adding phage. **a** Time to 0.1 OD for each phage treatment. Statistical significance determined with Mann–Whitney-U test with FDR multiple hypothesis correction. **b** Boxplot depicting measured CFUs/mL of *E. coli* or *S. enterica* at the end of growth. **c** Species ratios represented with frequency of *E. coli* at the end of growth. Ratios were calculated from plated CFUs. Statistical significance of **b**) and **c**) determined with Mann–Whitney-U test with FDR multiple hypothesis correction. ES = no phage controls, +P22*vir* (or +P22) = P22*vir* phage treatment, +T7 = T7 phage treatment

When the co-culture was exposed to the *S.* *enterica*-specific P22*vir* phage, P22*vir* rose to high titers (Supplementary Fig. 4), caused evolution of resistance in *S. enterica* (Table 1), and delayed community growth compared with no-phage controls (*p* = 0.0037, Fig. 2a). However, compared with no-phage controls, P22*vir* significantly affected neither the species densities of *E. coli* (*p* = 0.35, Fig. 2c) or *S. enterica* (*p* = 0.95, Fig. 2c), nor the frequency of *E. coli* (*p* = 0.51, Fig. 2b). Overall, P22*vir* infection of the co-culture delayed growth and did not change final community composition, as predicted by co-culture simulations.

**Table 1:** Resistance and mucoidy in phage-treated cooperative co-cultures

Treatment	No. of replicate communities	resistant^a^/total isolates	mucoid/total isolates
ES	10	0/30	0/30
ES + P22*vir*	5	15/15	0/15
ES + T7	3^b^	9/9	9/9

^a^Resistance assayed with cross-streaks

^b^Two replicate communities were driven extinct

The effects of T7 phage on the cooperative co-culture differed in some ways from the effects of P22*vir* phage. Like P22*vir*, T7 phage rose to high titers (Supplementary Fig. 4), caused evolution of resistance in its host - *E. coli* (Table 1), and delayed community growth compared with no-phage controls (*p* = 0.0037, Fig. 2a). However, there were changes to the cooperative co-culture composition. T7 infection of *E. coli* in a cooperative community decreased *E. coli* density in the presence of T7 (*p* = 0.017, Fig. 2b). In fact, *E. coli* went extinct in two of the five T7 phage replicate communities. In addition, the final frequency of *E. coli* relative to no-phage controls decreased following T7 phage attack (*p* = 0.008, Fig. 2c). Surprisingly, growth of *S. enterica* was not constrained by the phage-mediated death of *E. coli*. Instead, *S. enterica* reached between 8- and 35-fold higher density in the presence of T7 (Fig. 2c). In addition, *S. enterica* entered log-phase sooner, despite its dependence on *E. coli* secreted acetate (Supplementary Fig. 3a). This led to a rapid increase in biomass of communities with T7 phage (Supplementary Fig. 3b). Phage attack on *E. coli* appeared to release *S. enterica* from the constraints typically imposed on cross-feeding community partners—a novel result that we interrogated further.

### T7-resistant *E. coli* increase *S. enterica* densities in the absence of phage

**Fig. 3:**
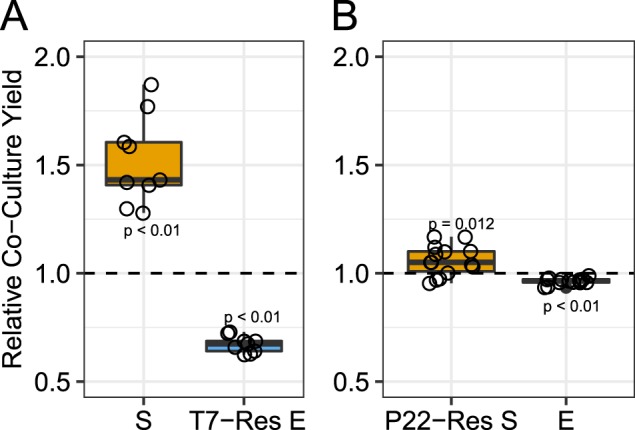
Relative yield of co-cultures with resistant isolates in the absence of phage. The relative yield (CFU) for each strain in co-cultures with one resistant partner as compared with the ancestral co-culture. **a** Relative yields of co-cultured ancestral *S. enterica* (S) and T7-resistant *E. coli* isolates (T7-Res E) (*n* = 9). **b** Relative yields of co-cultured P22*vir*-resistant *S. enterica* isolates (P22-Res S) and ancestral *E. coli* (E) (*n* = 15). Dashed lines represent standardized yields of ancestral *S. enterica* and *E. coli* co-cultures. Points represent averages of triplicate replicates. Statistical significance determined using Wilcox Sign Test with μ = 1

The increase of *S. enterica* during T7 infection of *E. coli* could result from T7-resistance altering *E. coli* secretion of cross-fed metabolites. To test if T7-resistant *E. coli* provide more metabolites than sensitive *E. coli*, we assayed the growth of *S. enterica* when paired with evolved *E. coli* isolates in the absence of phage. *S. enterica* reached an average of 1.43-fold higher density when co-cultured with evolved T7-resistant isolates than with ancestral *E. coli* (Fig. 3a, *p* = 0.004). In these co-cultures, the yield of T7-resistant *E. coli* only reached 67% of the yield of the ancestor (Fig. 3a, *p* = 0.004), suggesting that T7-resistant *E. coli* isolates supported a larger cross-feeding *S. enterica* population. We also paired P22*vir*-resistant *S. enterica* with ancestral *E. coli* and found that P22*vir* resistance led to an ~2% decrease in *E. coli* yield (Fig. 3b, *p* < 0.0005), and increased *S. enterica* 5% (Fig. 3b, *p* = 0.012). While our data illustrate that *S. enteric* receives more carbon from evolved *E. coli* than ancestral *E. coli*, we cannot differentiate between increased secretion or poor reuptake of carbon. These results suggest that the divergent impact of T7 and P22*vir* on community composition is in part driven by how resistance to each phage influences the abundance of cross-fed metabolites.

An alternative explanation for the asymmetric response to T7 and P22*vir* phage would be differences in the cost of resistance—such as decreased growth rates. However, costs of resistance to either T7 or P22*vir*, measured by isolate monoculture growth rate or species-specific co-culture growth rate, were less than 8% (Supplementary Fig. 2c). Furthermore, mathematical modeling suggested that costs of resistance, encoded by decreasing growth rate when phage resistant, do not alter final yields, but caused small delays in community growth (Supplementary Fig. 2a, b).

### *S. enterica* grows on cellular debris released by phage lysis

**Fig. 4:**
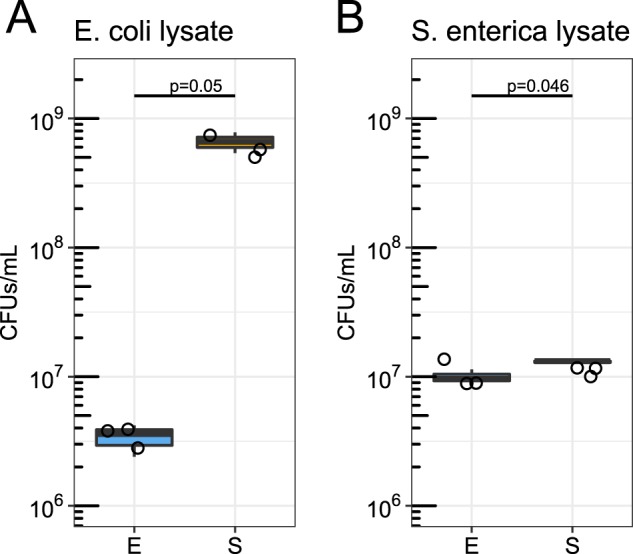
Growth of *S. enterica* and *E. coli* on sonicated cellular debris. *E. coli* (E) and *S. enterica* (S) monocultures were each grown in lactose minimal medium +25% (v/v) sonicated cellular debris lysate. **a** Monoculture yields on *E. coli* cellular debris lysate. **b** Monoculture yields on *S. enterica* cellular debris lysate. Cultures were inoculated with 5 × 10^5^ cells/ml. Statistical significance was tested with a Kruskal Wallis test

When lytic phage burst host cells to release phage progeny, intracellular carbon and nutrients are also released into the environment, referred to as the “viral shunt” [21]. We tested whether consumption of lysed *E. coli* cellular debris increased the density of *S. enterica* in the presence of T7 by measuring *E. coli* and *S. enterica* monoculture yields on cellular debris without phage. We produced cellular debris by sonicating monocultures of *E. coli* and *S. enterica* grown in minimal medium. We then grew ancestral *E. coli* or *S. enterica* in lysates in lactose minimal medium without methionine supplemented with 25% sonicated supernatant for 48 h without phage. We plated for CFUs to determine yields after 48 h of growth. While both *E. coli and S. enterica* were capable of growth in lactose minimal media supplemented with cellular debris, we observed different responses. *S. enterica* reached 100-fold higher densities than *E. coli* when both were grown independently on *E. coli* cellular debris (*p* = 0.050), and two-fold higher densities than *E. coli* when both were grown independently on *S. enterica* cellular debris (*p* = 0.046, Fig. 4). These results suggest that *S. enterica* more effeciently uses *E. coli* cellular debris released during T7 infection than *E. coli* uses *S. enterica* cellular debris released during P22*vir* infection. In fact, in lactose minimal medium supplemented with 25% sonicated cellular debris supernatant, *S. enterica* generated 7.3 new cells per cellular equivalent of *E. coli* cellular debris while *E. coli* generated 0.05 new cells per cellular equivalent of *S. enterica* cellular debris (Supplementary Table 4).

### A modified mathematical model incorporating changes in secretion profiles and cellular debris exchange does not reflect wet-lab experiments

**Fig. 5:**
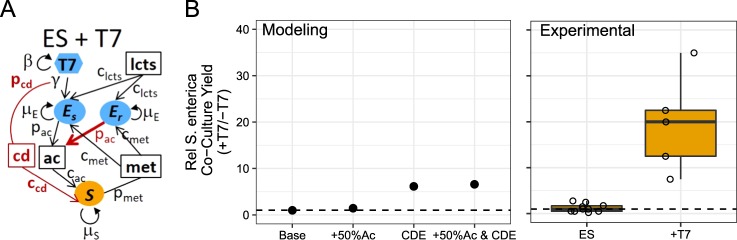
Mathematical model with increased acetate production and cellular-debris exchange increases final densities of *S. enterica* during *E. coli*-specific T7 phage attack. **a** Schematic of modified model of *E. coli*-specific T7 phage attack. Red text highlights the modifications of cellular debris (cd) and a 50% increase in acetate production (p_ac_) by T7-resistant *E. coli*. **b** Relative *S. enterica* co-culture yields of modeling (left panel) and experimental (right panel) results. Results are relative to no phage (−T7) control communities. Base = base model described in Fig. 1; +50%Ac = T7-resistant *E. coli* produce 50% more acetate compared with T7-sensitive *E. coli*; CDE = cellular debris exchange model where *E. coli* cells lysed by T7 generate cellular debris that can be used by *S. enterica*; +50%Ac & CDE = model combining increase in acetate production when *E. coli* is resistant to T7 phage and consumption of cellular debris by *S. enterica*

In wet-lab experiments, we observed that pairing T7-resistant *E. coli* with ancestral *S. enterica* in cooperative co-culture results in ~50% more *S. enterica* than when paired with ancestral *E. coli* (Fig. 3). We incorporated this into our model to test how changes in secretion profile changed *S. enterica* yields. We found that the increase in acetate production by resistant *E. coli* increased simulated *S. enterica* yields 1.45-fold compared with the base model (Fig. 5).

Wet-lab experiments also showed more efficient *S. enterica* growth on cellular debris than *E. coli* (Fig. 4). Therefore, we incorporated nonhost consumption of host cellular debris (cd) into our model as well. Cellular debris was included as a metabolite produced when host cells are lysed by phage (Fig. 5 and Supplementary Methods). One lysed *E. coli* host cell was set to generate enough nutrients for 7.33 *S. enterica* cells, in agreement with experimental measurements (Supplementary Table 4). Incorporating consumable cellular debris increased simulated *S. enterica* yields by 6.16-fold compared with the base model (Fig. 5).

Combining both increased acetate production and consumption of cellular debris mechanisms in the mathematical model increased the final density of *S. enterica* 6.60-fold, still far less than that observed experimentally (Fig. 5). In fact, we had to increase the production-to-consumption ratio to ~25 *S. enterica* produced per lysed *E. coli* cell, an unrealistic number, to match the observed increases in *S. enterica* yields in wet-lab experiments (Supplementary Fig. 5). Taken together, changes in carbon secretion and consumption of cellular debris partially explain the observed increases in *S. enterica* during T7 infection, but additional mechanisms likely contribute.

### *E. coli* evolves partial resistance to phage, T7, increasing *S. enterica* yields

**Fig. 6:**
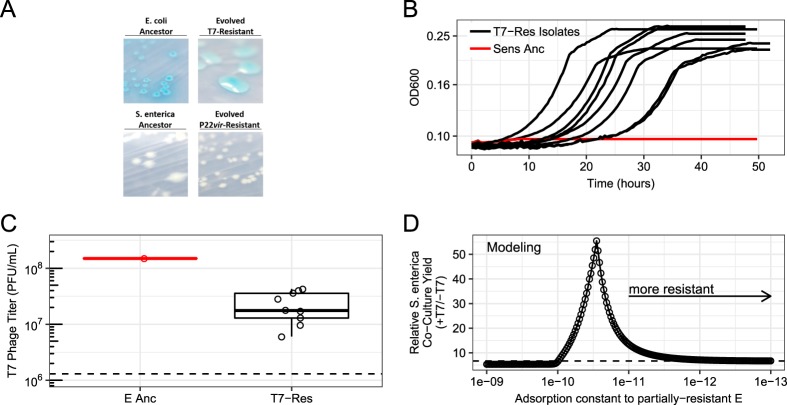
Experimental partial resistance quantification of *E. coli* mucoid T7-resistant and implications on nonhost *S. enterica* yield during modeled T7 phage attack. **a** Morphology comparison of representative *E. coli* T7-resistant isolate or representative P22*vir*-resistant *S. enterica* isolate and ancestral strains on lactose or acetate minimal media, respectively. **b** Representative growth curves of ancestral *E. coli* (Anc E, red line) or T7-exposed *E. coli* isolates (black lines) in the presence of T7 phage. Isolates were grown in lactose minimal medium supplemented with methionine with T7 phage (*n* = 9 isolates). **c** Titers of T7 phage recovered from infection of *E. coli* isolates. Isolates were inoculated at 0.01 OD and ~10^6^ T7 phage in lactose medium supplemented with methionine in triplicate. After culturing 72 h in 30 °C, phage lysates titered (*n* = 9 isolates, triplicate). Points are averages of three replicates. E Anc = Ancestral *E. coli*, T7-Res Isolates = mucoid evolved T7-resistant *E. coli* isolates. Dashed line = starting titer. **d** *S. enterica* co-culture yields with varying phage adsorption constants in the resistant *E. coli*. The mathematical model used had 50% increase in acetate production when *E. coli* is resistant to T7, the ability of *S. enterica* to consume cellular debris, and partial resistance of *E. coli* against T7 phage as determined by the adsorption factor. Dashed line = Relative *S. enterica* yield in same model with full* E. coli* resistance

*E. coli* isolates from replicate communities with T7 showed no inhibition of growth when cross-streaked against T7 phage (Table 1). In addition, evolved isolates grew in the presence of T7 while ancestral *E. coli* did not (Fig. 6b).

However, the mucoid phenotype of phage-resistant isolates suggested that partial resistance might contribute to the differential impact of T7 and P22*vir* phage on nonhost bacteria. T7 phage-resistant *E. coli* formed mucoid colonies, while P22*vir*-resistant *S. enterica* did not (Fig. 6a and Table 1). Mucoidy is frequently associated with partial resistance, providing incomplete protection against phage by decreasing efficiency of adsorption [26]. Finally, T7 phage increased more than ten-fold on mucoid *E. coli* isolates, much less than the 100-fold increase observed on ancestral *E. coli* isolates (Fig. 6c). This is qualitatively different from the full resistance observed in *S. enterica*. From an inoculum of 1 × 10^6^ PFU/mL, P22*vir* phage on average reached 6.3 × 10^8^ ± 3x10^8^ PFU/mL on ancestral *S. enterica* and 1.3 × 10^6^ ± 7.5x10^5^ PFU/mL on P22*vir*-resistant after 48 h of growth. Although growth of phage in evolved isolates may suggest T7 phage counter-adaptation, isolated ending phage populations and ancestral phage produced identical infection patterns when cross-streaked against evolved mucoid *E. coli* isolates (Supplementary Table 3). Furthermore, we note that similar phenotypes would be observed if a subset of the *E. coli* population reverted to the sensitive phenotype. However, it is unlikely that reversion instead of partial resistance would change the community-level interpretations we have suggested. These results suggest that evolved partially resistant *E. coli* isolates may continually be lysed throughout growth, increasing the total amount of cell debris available for *S. enterica* to consume.

Genome sequencing also supported partial resistance mechanisms in *E. coli* and a different mechanism in *S. enterica*. We whole-genome sequenced communities treated with either T7 or P22*vir* phage with Illumina sequencing and used *breseq* to identify mutations compared with reference genomes [27]. We focused on mutations unique to each phage treatment and >20% frequency in at least one replicate community. We identified two mutations in the intergenic region of the *clpx* and *lon* genes that reach high frequency in all T7-resistant *E. coli* populations (Table 2). Mutations in the *lon* gene encoding a protease of *E. coli* have been shown to cause mucoid phenotypes in *E. coli* [28]. We also identified a single mutation in four of five *S. enterica* genomes of P22*vir*-treated communities in a Gifsy-1 prophage terminase small subunit that rose to 80–90% (Table 2).

**Table 2:** Mutations identified by whole-genome sequencing of communities

Community Type	Genetic element	Mutation location	Mutation	Gene	Identified in no. of reps
ES + P22*vir*	*S. enterica* genome	Coding region (486/495bp)	Frameshift (+G)	STM2609—DNA packaging-like protein, small terminase subunit, in *Gifsy-1* prophage	4/5
ES + T7	*E. coli* genome	Intergenic (+118/−67)	Δ5 bp:: repeat_region(+)+4 bp	*clpX—lon* intergenic region, upstream of *lon*	1/3
	*E. coli* genome	Intergenic (+90/−93)	repeat_region (+)+6 bp:: Δ1 bp	*clpX—lon* intergenic region, upstream of *lon*	3/3

Finally, we leveraged our model to assess the impact of *E. coli* partial resistance to T7 phage on yield of the nonhost, *S. enterica*. We incorporated partial resistance by decreasing the T7 adsorption constant to reduce the frequency of successful phage infection of partially resistant *E. coli* which results in host cells lysing and releasing cellular debris throughout growth. Adding partial resistance, in addition to increasing acetate production of T7-resistant *E. coli* and allowing *S. enterica* to consume *E. coli* cellular debris, led to a maximum 55.5-fold increase in *S. enterica* yield, which is greater than the observed wet-lab increases of *S. enterica* (Fig. 6d). Furthermore, intermediate adsorption values corresponding to partial resistance phenotypes led to the highest yields of *S. enterica* (Fig. 6d). These results suggest that multiple mechanisms including increased acetate production, consumption of phage-released cellular debris, and partial phage-resistance mechanisms all led to the increase in the final yield of *S. enterica* during T7 infection of *E. coli*.

## Discussion

In summary, our results suggest that lytic phage can dramatically impact communities of cooperating cross-feeding bacteria by changing yields of nonhost species through multiple mechanisms. Previously published literature about responses to abiotic stressors led us to initially predict that attack of a host species with lytic phage would suppress the cross-feeding community. However, our resource-explicit models instead indicated that resistance could quickly evolve, leading to a temporary delay in community growth before ultimately reaching similar yields and species ratios to those observed in communities not attacked by phage (Fig. 1). In agreement with the mathematical model, attack of *S. enterica* with P22*vir* phage delayed community growth in wet-lab experiments and had little effect on final species ratios (Fig. 2 and Supplementary Fig. 3). In contrast, *E. coli*-specific T7 attack dramatically altered the final species ratios in favor of *S. enterica*, the nonhost, but caused a relatively small delay in community growth (Fig. 2 and Supplementary Fig. 3). Experimental and mathematical results suggest that a combination of several factors contributed to the increase of *S. enterica* in the presence of T7. These factors included changes in the extent of cross-feeding by resistant *E. coli* (Figs. 3 and 5), growth on cellular debris released from phage-lysed cells (Figs. 4 and 5), and the evolution of partial resistance in *E. coli* (Fig. 6). Our modeling suggests that divergent community responses to phage can be accurately predicted by incorporating metabolic mechanisms and the level of phage resistance.

We observed that the impact of phage extended beyond the targeted host to the other member of a cross-feeding pair. *P22vir* infection delayed growth of the nonhost, while T7 infection facilitated growth of the nonhost. In both cases the effect on growth of nonhosts was indirect and was mediated by changes in the metabolites available in the system. The *P22vir* results demonstrate that cross-feeding metabolic dependencies can make the entire community susceptible to perturbations of a single species, similar to previous findings with antibiotic and genetic perturbations [18, 19], and highlights the dangers of a cross-feeding lifestyle [29]. However, here we demonstrate that evolution of phage resistance can rapidly return cross-feeding co-cultures to the unperturbed state. In contrast, T7 had qualitatively distinct impacts on the community that were mediated by consumption of cellular debris. Release of nutrients through cell lysis is likely to generate indirect effects on species abundance independent of microbial interactions. Indeed, viral lysis of bacteria is thought to play a major role in shaping the composition of diverse microbial communities [1, 2, 4]. Phage are frequently touted as tools for targeted treatment of pathogenic bacteria infections [7, 30]. However, our results suggest that even strain-specific phage can have broader impacts on microbial communities, which could lead to diverse phage therapy outcomes.

We predict that an understanding of what metabolites mediate cross-feeding will make predicting indirect effects of phage more accurate. We have shown two contrasting effects of phage attack on a cross-feeding microbial community. One major reason for the divergent effects is likely the identity of the cross-fed nutrients. In our microbial community, *S. enterica* receives acetate from *E. coli*, while *E. coli* receives methionine from *S. enterica*. Bennett et. al. showed that intracellular pools of carbon compounds are larger than intracellular pools of methionine [31]. Furthermore, it may be easier for cells to scavenge carbon from biomass components than methionine from proteins. Taken together, it is likely that *S. enterica* has easier access to required metabolites in cellular debris than *E. coli* because *S. enterica* reached higher densities on multiple cellular debris types than *E. coli* (Fig. 4 and Supplementary Table 4). In addition, the nutritional quality of cellular debris appears to vary, as the difference between *S. enterica* and *E. coli* yields on cellular debris was larger on *E. coli* debris than on *S. enterica* debris (Fig. 4 and Supplementary Table 4). We acknowledge that the metabolites released by phage lysis are likely different from those released by sonication of uninfected cells due to phage-mediated host metabolism changes [32]; however, it is unlikely that these differences would qualitatively alter our results. These results suggest that metabolic mechanisms play a critical role in determining the impact of phage in cross-feeding systems and highlight the need for further methods to quantitatively incorporate these mechanisms in our models [15].

In addition, the magnitude of the community response to T7 phage was influenced by the mechanism of phage resistance that evolved. Both mutations identified in *E. coli* genomes exposed to T7 phage were upstream of the *lon* gene encoding the lon protease, a mutation consistent with a partial resistance phenotype [28]. Down-regulation of the lon protease has been found to cause a mucoid phenotype [28] and negatively regulates the activator of capsular genes *rscA* [33]. Qimron et al. showed that knocking out *rscA* also caused mucoidy and partial phage resistance against four phage, including T7 [34]. Our model suggests that *E. coli*’s incomplete resistance significantly increased the indirect effects of *E.* *coli*-specific T7 phage on *S. enterica*. If complete resistance was modeled, phage rapidly killed sensitive cells generating only a small pool of cellular debris. In contrast, partially resistant *E. coli* continued to lyse (though at a lower rate) and generate cell debris throughout growth. This continual lysis substantially increased yields of the nonhost *S. enterica*. Partial resistance should also allow the phage population to be maintained and should therefore lead to lasting changes in species ratios barring further evolution by phage or host bacteria. In conclusion, evolution of partial resistance to phage infection has the potential to generate lasting changes in community composition due to the continual generation of consumable cellular debris.

Understanding the indirect impacts of phage in microbial communities is critical as we strive to manage microbial ecosystems. Four recent studies using phage therapy in mouse models also observed extensive indirect effects of phage in microbial communities [35–38]. All four studies reported that phage therapy changed abundances of nonhost genera in mouse digestive tracts; however, no mechanisms were confirmed. Understanding how and why phage influence microbial communities is important for controlling bacteria populations, particularly in the food industry and medical field [39, 40]. We conclude that understanding the ways that bacteria interact, the ability of species to use nutrients from lysed bacteria, and the extent of phage resistance are paramount for predicting the effects of phage attack in diverse microbial communities.

## Material and methods

### Mathematical simulations

We used resource-explicit ordinary differential equation models to simulate cooperative growth of *E. coli*, and *S. enterica* (Supplementary Methods). Growth of the bacterial species was governed by Monod kinetics with multiplicative limitation for resources. Production of cross-fed nutrients was growth dependent. Our base model without phage infection used two equations to directly track *E. coli* (E) and *S. enterica* (S):

$$\frac{{dE}}{{dt}} = E \times \mu _E \times \frac{{\mathrm{lcts}}}{{\mathrm{lcts} + k_{E_{\mathrm{lcts}}}}} \times \frac{{\mathrm{met}}}{{\mathrm{met} + k_{E_{\mathrm{met}}}}}$$

$$\frac{{dS}}{{dt}} = S \times \mu _S \times \frac{{ac}}{{ac + k_{S_{ac}}}},$$

where E or S is the bacterial population size, μ_x_ is a species-specific growth rate (h^−1^), k_x_ is a species- and metabolite-specific Monod constant, and lcts, met, and ac represent concentrations of lactose, methionine, and acetate in g/200μl to compare with wet-lab results.

We added equations for *E. coli*-specific T7 or *S. enterica*-specific P22*vir* lytic phage infection (Fig. 1). For example, sensitive *E. coli* (Es) and T7 phage interacted through the following equations:

$$\frac{dEs}{dt} = \;\, Es \times \mu _{E} \times \frac{\mathrm{lcts}}{\mathrm{lcts} + k_{E_{\mathrm{lcts}}}} \times \frac{\mathrm{met}}{\mathrm{met} + k_{E_{\mathrm{met}}}}\\ - \left(Es \times T7 \times {\mathrm{adsorption}}\,\,{\mathrm{constant}}\right)$$

$$\frac{dT7}{dt} = T7 \times {\mathrm{burst}} \times Es \times {\mathrm{adsorption}}\,\,{\mathrm{constant}}$$

Models contain phage-sensitive (E_s_ or S_s_) host strains, phage-resistant (E_r_ or S_r_) host strains, or nonhost (E or S) strains as needed. Phage growth was restricted to occur only on growing cells by adding a constraint that if there were no resources, phage could not replicate (see Supplementary Methods for details). In a second model, we added an equation for cellular debris (cd) produced when sensitive host cells were killed by phage. The cellular debris was consumed by nonhost species (Fig. 5). We altered metabolite production by changing the production parameters (p_x_) and encoded partial resistance by changing the adsorption rate parameter (γ). Our model assumes that resistant hosts were present at the beginning of the community growth and were seeded at 0.1% of the host population. All simulations were run in R with the DeSolve package, using the LSODA solver [41]. Refer to Supplementary Figures and Methods and Supplementary Table 1 for details.

### Ancestral bacterial co-culture system and viral strains

Ancestral *E. coli* and *S. enterica* strains are previously described. Briefly, the ancestral *E. coli* K12 BW25113 *metB::kan* is a methionine auxotroph from the Keio collection with the lac operon restored. *S. enterica* LT2 was evolved to secrete methionine [25]. *E. coli* is tagged with a cyan fluorescent protein-encoding gene integrated at the attB lambda integration site and driven by a constitutive lambda promoter. *S. enterica* is tagged with a yellow fluorescent protein encoding gene under the same promoter and at the same integration site. Co-cultures were grown in lactose hypho minimal medium (5.84 mM lactose, 7.26 mM K_2_HPO_4_, 0.88 mM NaH_2_PO_4_, 1.89 mM [NH_4_]_2_SO_4_, 0.41 mM MgSO_4_) [42]. Monocultures of *E. coli* were supplemented with 80 μM of l-methionine and monocultures of *S. enterica* replaced lactose with acetate. T7 phage is an *E. coli*-specific lytic bacteriophage and P22*vir* phage is a *S. enterica*-specific lytic bacteriophage. Virus stocks were provided by I. J. Molineaux and were stored at −80 °C. Working stocks of phage were grown on ancestral *E. coli* or *S. enterica* cultures in minimal medium and stored at 4 °C.

### Microbial community growth

To measure bacteria community growth, mid-log cultures started from a single bacterial colony were used to inoculate 200μl of medium in a 96-well plate with 10^5^ cells for each bacterial species per well, and 10^2^ total phage (MOI = 0.01) where indicated (Supplementary Table 2). The 96-well plates were incubated in a Tecan Pro200 plate reader for 96–120 h at 30 °C with shaking. OD600, *E. coli*-specific CFP (Ex: 430 nm; Em: 490 nm), and *S. enterica*-specific YFP (Ex: 500 nm; Em: 530 nm) fluorescence were read every 20 min. Fluorescent protein signals were converted to species-specific OD equivalents using an experimentally-determined conversion factor as described [19]. Five phage treatments and five controls were used in each experiment. Phage were tested in separate experiments totaling five T7 treatments, five P22*vir* treatments, and 10 no-phage controls. Following growth, we plated for CFUs of both bacterial species—*E. coli* on lactose minimal medium plates with excess methionine and *S. enterica* on citrate minimal medium. X-gal (5-bromo-4-chloro-3-indolyl-β-D-galactopyranoside) in plates differentiated between *E. coli* and *S. enterica*. Phage population sizes were measured by plating for plaques on LB with 0.3% LB top agar with a lawn of the ancestral, sensitive host. Phage plates were incubated at 37 °C and bacterial plates at 30 °C.

### Testing for evolution of phage resistance: cross-streaking assays

Three colonies per replicate community were isolated on minimal media plates. Resistance to ancestral phage was tested with cross-streaking assays on minimal media plates. Phage stock (~10^8^ PFU) was spread in a line across a plate, bacterial isolates were streaked perpendicular to the phage culture, and plates were incubated at 30 °C for 24–72 h. Bacterial isolates with clearing around the phage streak were deemed sensitive and isolates with no clearing were resistant.

### Phenotyping phage resistant isolates

Isolates were cultured alone or in cooperative co-culture as indicated in minimal medium in a 96-well plate. Bacteria were inoculated at 10^5^ cells per well. OD600, and CFP or YFP fluorescence were recorded with the TecanPro200 shaking plate reader for 72 h. Growth rates were calculated by fitting Baranyi growth curves [43] to fluorescent protein data transformed into OD-equivalents (see above) and compared with ancestral strains grown in either monoculture or cooperative co-culture.

### Testing phage-mediated cellular debris exchange

*E. coli* was grown in lactose + methionine minimal medium and *S. enterica* was grown in acetate minimal medium. After the stationary phase was reached (OD_600_ ~0.5), cells were pelleted, sonicated (10, 30 s pulses), and filter sterilized with a 0.22 μm filter. Filtered sonication supernatants were checked for sterility by plating. Ancestral bacteria were inoculated at 5 × 10^5^ cells/ml in lactose minimal medium supplemented with 25% filtered sonication supernatant and incubated at 30 °C for 48 h. Cultures were plated to enumerate CFUs.

### Whole-genome sequencing of communities and analysis

Communities were inoculated from frozen stocks into lactose minimal media and grown at 30 °C for 4 days. Total community DNA was isolated using the Zymo Quick-gDNA Miniprep Kit (11-317C). Illumina sequencing libraries were prepared according to the Nextera XT DNA Library Prep Kit protocol, submitted to the University of Minnesota Genomics Center for QC analysis, and sequenced on an Illumina Hi-Seq with 125 bp paired-end reads. We used the *breseq* tool version 0.28.1 [27] to align Illumina reads to the following reference genomes: *E. coli str. K-12 substr. MG1655* (Accession No: NC_000913.3), *S. enterica subsp. enterica serovar Typhimurium str. LT2* (Accession No: NC_003197.2, NC_003277.2), Enterobacteria phage T7 (Accession No: NC_001604.1), and Enterobacteria phage P22 (Accession No: NC_002371.2) and predict polymorphisms (-p command). Mutations lists for resistant populations were filtered to remove mutations common between ancestral strains and reference genomes. We kept mutations that were unique to each phage treatment and arose in populations >20% (Table 2).

## Supplementary information

An Ordinary Differential Equation Model Exploring Phage Effects on a Cross-Feeding Microbial Co-culture Community in R
