# Supplementary material for: Lytic bacteriophage have diverse indirect effects in a synthetic cross-feeding community
Source: ISME J. 2019 Oct 2;14(1):123–34. doi: 10.1038/s41396-019-0511-z (PMC6908662; doi:10.1038/s41396-019-0511-z)
Supplement: Supplementary file 1 — An Ordinary Differential Equation Model Exploring Phage Effects on a Cross-Feeding Microbial Co-culture Community in R [file 41396_2019_511_MOESM1_ESM.docx]

Supplementary Figures and Methods

An Ordinary Differential Equation Model Exploring Phage Effects on a

Cross-Feeding Microbial Co-culture Community in R

**Lisa Fazzino^1,4^, Jeremy Anisman^2,4^, Jeremy M. Chacón^3,4^, Richard H. Heineman^5^, William R. Harcombe*^1,3,4^**

*^1^Department of Microbiology and Immunology, University of Minnesota, Minneapolis, MN, USA;*

*^2^College of Continuing and Professional Studies, University of Minnesota, Minneapolis, MN, USA;*

*^3^Ecology, Evolution, and Behavior, University of Minnesota, Minneapolis, MN, USA;*

*^4^BioTechnology Institute, University of Minnesota, Minneapolis, MN, USA;*

*^5^Biology Department, Kutztown University, Kutztown, PA, USA*

1. Resource-Explicit ODE Model of Phage Infection of a Bipartite Cross-Feeding Microbial Community

We used the following systems of ordinary differential equations to model changes in biotic populations of an engineered mutualistic co-culture community comprised of *Escherichia coli* (E) and *Salmonella enterica* (S)[1]. Briefly, growth of bacteria followed Monod dynamics and phage, when present, had a linear effect on host bacteria. Depending on the hypothesis being tested, we added equations to model the effects of E-specific phage (T7) and S-specific phage (P22*vir* or P22) (see below). Replication of phage populations depended on whether host bacterial populations were actively replicating. Bacterial populations were comprised of phage-sensitive (s) and phage-resistant (r) individuals, indicated by a lowercase letter. The model used the units of cells/200μL and g/200μL for nutrient resources. Definitions of model parameters are in Supplementary Table 1. Parameters include phage population parameters, nutrient uptake, and conversion parameters. Although we used parameter values and variable names that mimic the *E. coli / S. enterica* wet-lab community, these models are generalizable to any species and phage system.

1.1 Base Monod Growth Model Without Phage Infection

We started with the following set of equations as a base model of *E. coli* and *S. enterica* cross-feeding interactions in lactose minimal medium without phage infection. In this base model, resistant bacteria were not considered.

Biotic Equations:

$$\mu_{E, relative} =\left( \frac{lcts}{lcts+ k_{Elcts}} \right)\left( \frac{met}{met+ k_{Emet}} \right)$$

$$\frac{dEs}{dt} =\left( Es \right)\left( \mu_{Es} \right)(\mu_{E, relative})$$

$$\frac{dS}{dt} = (S)(\mu_{S})\left( \frac{ac}{ac+ k_{Sac}} \right)$$

Example Abiotic Equations:

$$\frac{dlcts}{dt}= -\left( Es \right)\left( \mu_{Es} \right)(\mu_{E, relative})*c_{Elcts}$$

$$\frac{dmet}{dt}= -\left( Es \right)\left( \mu_{Es} \right)\left( \mu_{E, relative} \right)*c_{Emet}+ (S)(\mu_{S})\left( \frac{ac}{ac+ k_{Sac}} \right)p_{met}$$

where $\mu_{x}$represent isolate-specific growth rates. In this model formulation, *E. coli* growth is limited by lactose (lcts) and methionine (met) concentrations in a multiplicative manner, and saturates with Monod half-saturation parameter (k_x_). Accumulation of produced metabolites is growth dependent.

1.2 Modelling Phage Infection of Cross-Feeding Bacterial Community

We added phage infection to the model equations by including a phage equation, a resistant host equation with a potentially different maximum growth rate parameter (μ_Er_), and a term in the host to represent phage-mediated death. Phage-mediated death is modeled using a classic, linear, host-parasite interaction. However, since T7 phage reproduction significantly decreases on stationary phase cells [2], in our model, phage only produced new progeny from actively growing hosts (see example cases in E growth equations below).

The following example set of equations model T7 phage infection of *E. coli:*

Biotic Equations:

$$\mu_{E, relative} =\left( \frac{lcts}{lcts+ k_{Elcts}} \right)\left( \frac{met}{met+ k_{Emet}} \right)$$

$$\frac{dEs}{dt} =\left\{ \begin{aligned} \left( Es \right)\left( \mu_{Es} \right)\left( \mu_{E, relative} \right)- Es \left( T7 \right)\left( \gamma\right), if(\mu_{E, relative})>0.0001 \\ \left( Es \right)\left( \mu_{Es} \right)(\mu_{E, relative}), otherwise \end{aligned} \right.$$

$$\frac{dEr}{dt} =\left\{ \begin{aligned} \left( Er \right)\left( \mu_{Er} \right)\left( \mu_{E, relative} \right)- Er \left( T7 \right)\left( \gamma_{partial} \right), if(\mu_{E, relative})>0.0001 \\ \left( Er \right)\left( \mu_{Er} \right)(\mu_{E, relative}), otherwise \end{aligned} \right.$$

$$\frac{dS}{dt} = (S)(\mu_{S})\left( \frac{ac}{ac+ k_{Sac}} \right)$$

$$\frac{dT7}{dt} =\left\{ \begin{aligned} \left( T7 \right)\left( \beta\right)\left( Es \right)\left( \mu_{Es} \right)\left( \mu_{E, relative} \right)\left( \gamma\right)+ \\ \left( T7 \right)\left( \beta\right)\left( Er \right)\left( \mu_{Er} \right)\left( \mu_{E, relative} \right)\left( \gamma_{partial} \right), if(\mu_{E, relative})>0.0001 \\ 0, otherwise \end{aligned} \right.$$

Example Abiotic Equations:

$\frac{dlcts}{dt}= -\left( Es \right)\left( \mu_{Es} \right)\left( \mu_{E, relative} \right)*c_{Elcts}- \left( Er \right)\left( \mu_{Er} \right)\left( \mu_{E, relative} \right)*c_{Elcts}$

$\frac{dmet}{dt}= -\left( Es \right)\left( \mu_{Es} \right)\left( \mu_{E, relative} \right)*c_{Emet}- \left( Er \right)\left( \mu_{Er} \right)\left( \mu_{E, relative} \right)*c_{Emet}+ (S)(\mu_{S})\left( \frac{ac}{ac+ k_{Sac}} \right)p_{met}$

where $Es (T7)(\gamma)$ represents the number of sensitive *E. coli* host cells killed by T7 phage infection with the adsorption constant ($\gamma$), $\beta$ is the phage burst size, and Er represents the phage-resistance genotype that is seeded in at 0.1% of the sensitive population level. In simulations where Er was partially-resistant but still able to be infected by T7 phage, $\gamma_{partial}$ > 0, otherwise $\gamma_{partial}$ = 0.

To model P22*vir* infection of *S. enterica*, similar equations as above were used with Ss, Sr, and P22 equations to replace Es, Er, and T7 equations.

2. Resource-Explicit ODE Model Including Phage-Lysis Mediated Exchange of Cellular Debris

To our base model, we added the ability of *S. enterica* cells to consume cellular debris released during phage lysis of host cells. To accomplish this, we added a metabolite equation to represent cellular debris (cd). Production of cellular debris occurred when *E. coli* hosts died by phage lysis, with production rate parameter p_e_cd. Non-hosts (S) could grow using cellular debris with Monod kinetics. The consumption parameter c_s_cd describes the amount of cellular debris required to produce an *S. enterica* cell from cellular debris. *S. enterica* cells growing on acetate and cellular debris could grow faster than *S. enterica* cells growing on either resource alone because acetate and cellular debris could be used as substitutes for each other. Furthermore, growth on both substrates was additive. Note that, similar to above, usually *Er* were fully resistant and did not produce cellular debris (i.e. $\gamma_{partial}$=0).

$$\frac{dcd}{dt} =\left\{ \begin{aligned} Es \left( T7 \right)\left( \gamma\right)\left( p_{cd} \right)+Er \left( T7 \right)\left( \gamma_{partial} \right)\left( p_{cd} \right)- \\ \left( S \right)\left( \mu S \right)\left( \frac{cd}{cd+ k_{Scd}} \right) * c_{cd}, if(\mu_{E, relative})>0.0001 \\ - \left( S \right)\left( \mu S \right)\left( \frac{cd}{cd+ k_{Scd}} \right) * c_{cd}, otherwise \end{aligned} \right.$$

$$\frac{dS}{dt} = (S)(\mu S)\left( \frac{ac}{ac+ k_{Sac}} \right)+ (S)(\mu S)\left( \frac{cd}{cd+ k_{Scd}} \right)$$

3. Model Parameters

Table of model parameters with references can be found in Supplementary Table 1.

4. Simulation Framework

ODE models were numerically integrated using the lsoda solver from the deSolve package in R. Time was in units of hours. Code to run a sample model with a subset of parameters is included in the supplementary R script below.

References

[1] Harcombe WR. Novel cooperation experimentally evolved between species. Evolution. 2010;64:2166–72.

[2] Yin, J. A quantifiable phenotype of viral propagation. Biochem. Biophys. Res. Comms. 1991;174,2:1009-1014.

[3] Harcombe WR, Riehl WJ, Dukovski I, Granger BR, Betts A, Lang AH, et al. Metabolic resource allocation in individual microbes determines ecosystem interactions and spatial dynamics. Cell Rep. 2014;7:1104–15.

[4] De Paepe M and Taddei F (2006) Viruses' Life History: Towards a Mechanistic Basis of a Trade-Off between Survival and Reproduction among Phages. PLoS Biology 4(7): e193

# Supplementary R Script

# Resource-explicit base mathematical model for Fazzino et al. 2019.

# Set working directory with setwd()

library(deSolve)

library(dplyr)

library(tidyr)

# this model includes all possible interactions and both phage.

model_with_resources = function(t,n,parms){

with(as.list(c(t,n,parms)),{

# We first keep bacteria and metabolites from getting so rare that solver errors occur.

if (Es < 1e-20){

Es = 0

}

if (Er < 1e-20){

Er = 0

}

if (Ss < 1e-20){

Ss = 0

}

if (Sr < 1e-20){

Sr = 0

}

if (lcts < 1e-50){

lcts = 0

}

if (ac < 1e-50){

ac = 0

}

if (guts < 1e-50){

guts = 0

}

# Calculate the proportion of max growth rate bacteria should have given resource levels

relative_mu_E = (lcts / (lcts + k_e_lcts)) * (met / (met + k_e_met))

relative_mu_S = ac / (ac + k_s_ac)

# next, calculate actual growth

birth_Es = Es * mu_es * relative_mu_E

birth_Er = Er * mu_er * relative_mu_E

birth_Ss_eating_guts = Ss * mu_s * (guts / (guts + k_s_guts))

birth_Ss_eating_ac = Ss * mu_s * relative_mu_S

birth_Sr_eating_guts = Sr * mu_s * (guts / (guts + k_s_guts))

birth_Sr_eating_ac = Sr * mu_s * relative_mu_S

birth_Ss = birth_Ss_eating_guts + birth_Ss_eating_ac

birth_Sr = birth_Sr_eating_guts + birth_Sr_eating_ac

# to figure out impact of phage we test if bacteria have enough resources

# metabolic_threshold set at 0.0001

if (relative_mu_E <= metabolic_threshold){

death_Es = 0

death_Er = 0

birth_T7 = 0

dT7 = birth_T7

}else{

death_Es = Es*T7*T7ads

death_Er = Er*T7*T7part_r_ads

birth_T7 = T7*T7burst*Es*T7ads + T7*T7burst*Er*T7part_r_ads

dT7 = birth_T7

}

# apply a similar strategy for death of S and birth of P22

if (relative_mu_S <= metabolic_threshold){

death_Ss = 0

death_Sr = 0

birth_P22 = 0

dP22 = birth_P22

}

else{

death_Ss = Ss*P22*P22ads

death_Sr = 0 # no partial resistance in Sr

birth_P22 = Ss*P22burst*P22*P22ads

dP22 = birth_P22

}

# now we calculate the actual dN / dt for Es and Er and Ss and Sr

dEs = birth_Es - death_Es

dEr = birth_Er - death_Er

dSs = birth_Ss - death_Ss

dSr = birth_Sr - death_Sr

# finally, we explicitly track dN/dt for each of the resources

#consumption

consumed_lcts = (birth_Es + birth_Er) * c_e_lcts

consumed_met = (birth_Es + birth_Er) * c_e_met

consumed_ac = (birth_Ss_eating_ac + birth_Sr_eating_ac) * c_s_ac

consumed_guts = (birth_Ss_eating_guts + birth_Sr_eating_guts) * c_s_guts

#production

produced_lcts = 0

produced_met = (birth_Ss + birth_Sr) * p_s_met

# Er can sometimes produce a different amount of met

produced_ac = (birth_Es * p_es_ac) + (birth_Er * p_er_ac)

produced_guts = (death_Es + death_Er) * p_e_guts

dlcts = produced_lcts - consumed_lcts

dmet = produced_met - consumed_met

dac = produced_ac - consumed_ac

dguts = produced_guts - consumed_guts

list(c(dEs, dEr, dSs, dSr, dT7, dP22, dlcts, dmet, dac, dguts))

})

}


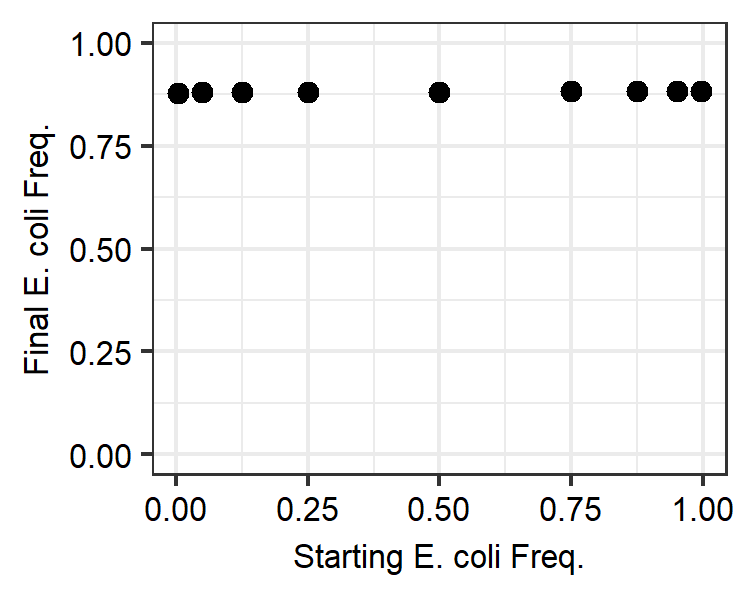


**Supplementary Figure 1. Species frequencies converge in simulated growth regardless of starting frequencies.** We began the simulation with various *E. coli* and *S. enterica* frequencies. Total beginning biomass was 2 x 10^6^ cells regardless of starting ratio. We simulated cooperative growth until all lactose was consumed. Final *E. coli* frequencies of the total bacterial population (E + S) were calculated. Frequencies converged to ~0.88 (88% *E. coli*).

**
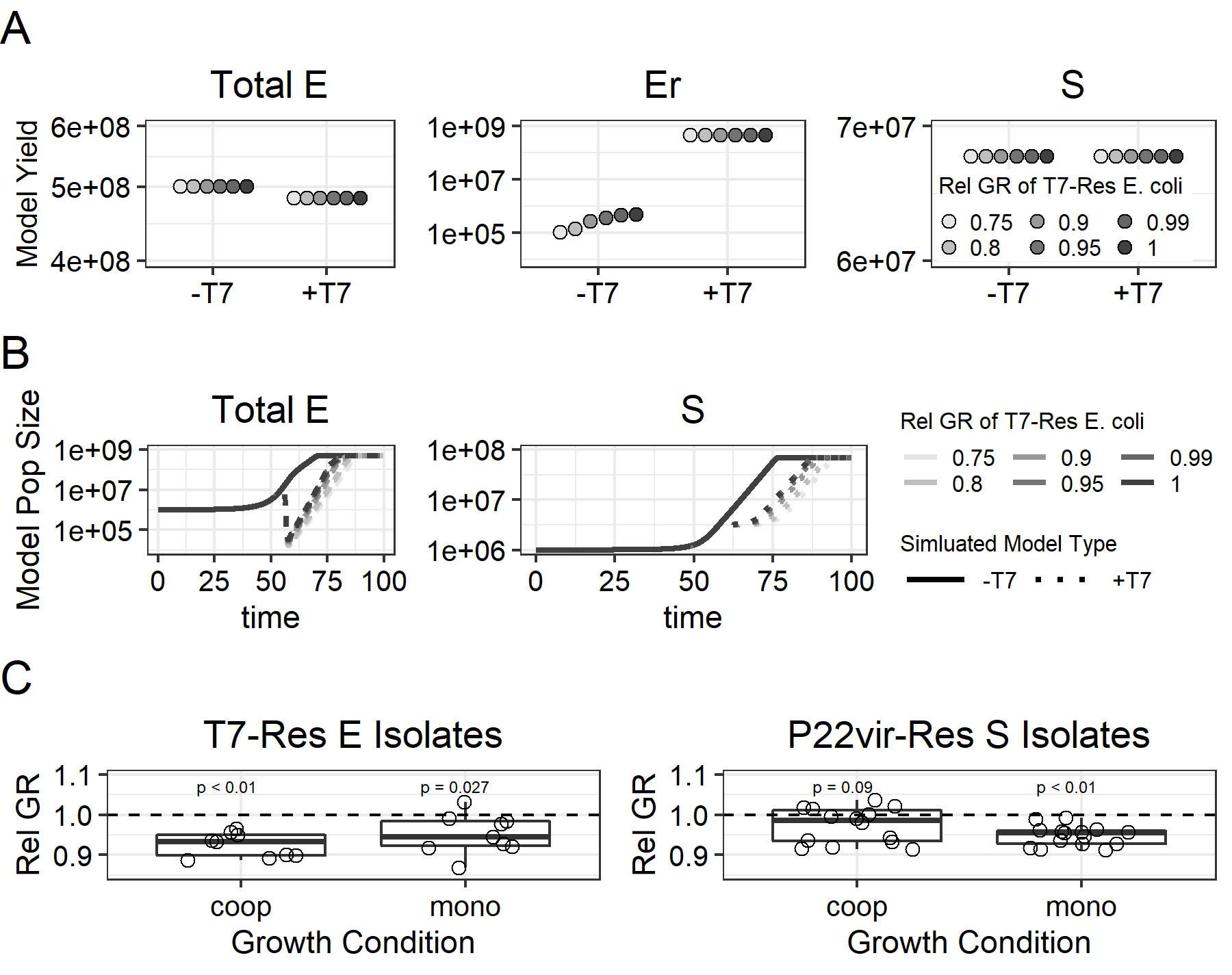
**

**Supplementary Figure 2. Simulated and measured costs of resistance do not qualitatively change growth dynamics.** In mathematical models, costs of resistance were simulated by decreasing the maximum growth rate of T7-resistant *E. coli* genotypes. Costs varied from 100% maximum relative growth rate (0% cost) to 75% maximum relative growth rate (25% cost). Simulations were run with and without phage. Costs did not change **A)** simulated yields of *E. coli* or *S. enterica*, but slightly alter final yields of resistant *E. coli* in cooperative co-cultures without phage. **B)** Costs also caused small growth delays. **C)** In wet-lab experiments, costs of resistance were measured for isolates by comparing growth rates in monoculture or co-culture with the ancestral isolate or co-cultured pair. Measured growth rates were standardized to ancestral growth rates. Average costs ranged from 0%-8% depending on the host species and growth conditions. Statistical significance determined with a one-sided T-test with mu = 1.


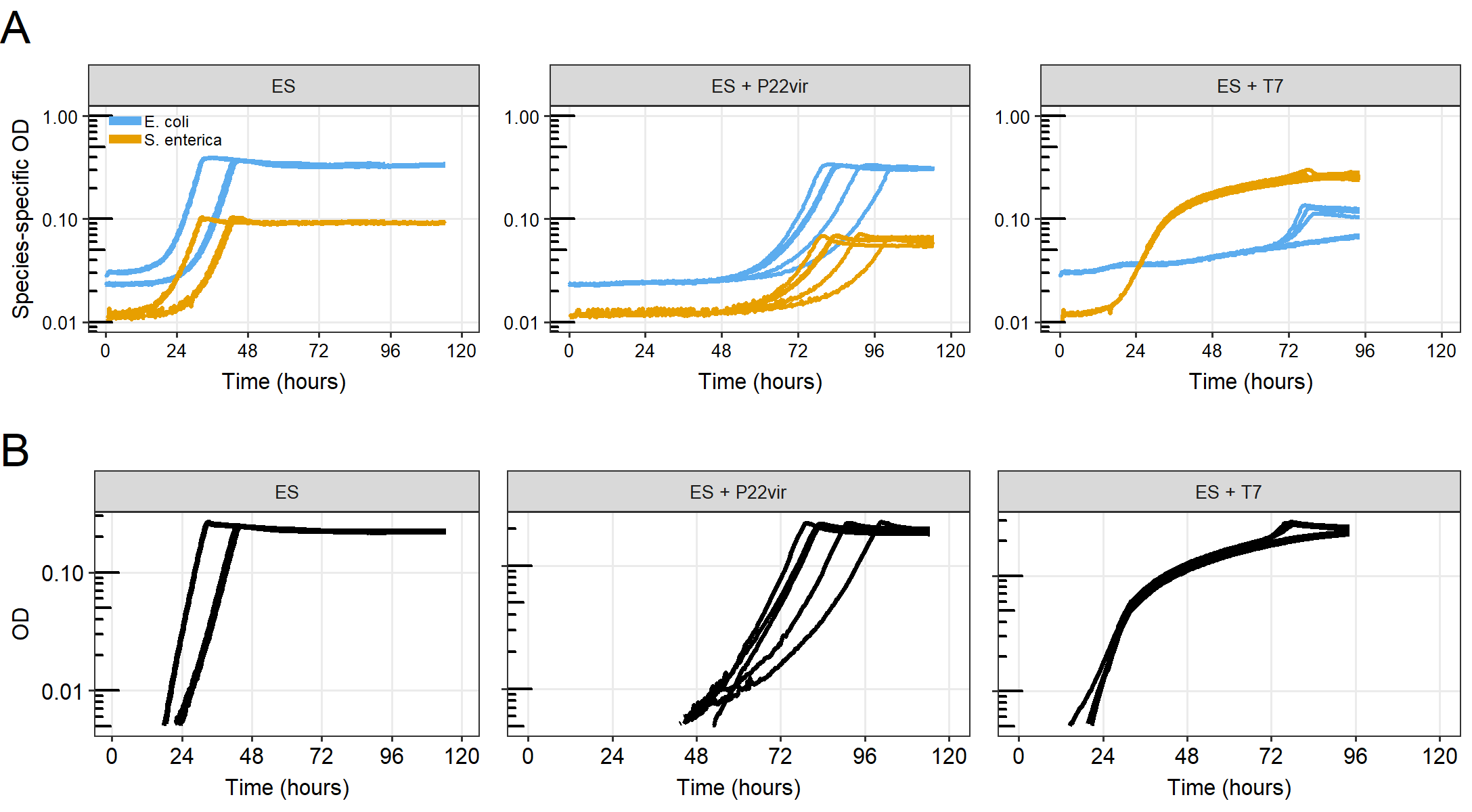


**Supplementary Figure 3. Community OD shows more delayed growth during P22*vir* attack than T7 attack. A)** Species-specific growth curves calculated by transforming fluorescence into species-specific OD for three treatments: no phage (ES), *S. enterica*-specific P22*vir* phage (ES + P22), or *E. coli*-specific T7 phage (ES + T7). *E. coli* (CFP) = blue, *S. enterica* (YFP) = yellow. Note: In ES + T7 panel (right), lower blue lines are two communities in which *E. coli* went extinct. CFP is not zero in these communities due to bleed-through from YFP channel. **B)** OD growth curves of cooperating communities treated with the indicated phage were measured every 20min in a shaking plate reader. OD is a proxy for growth of the whole bacterial community.


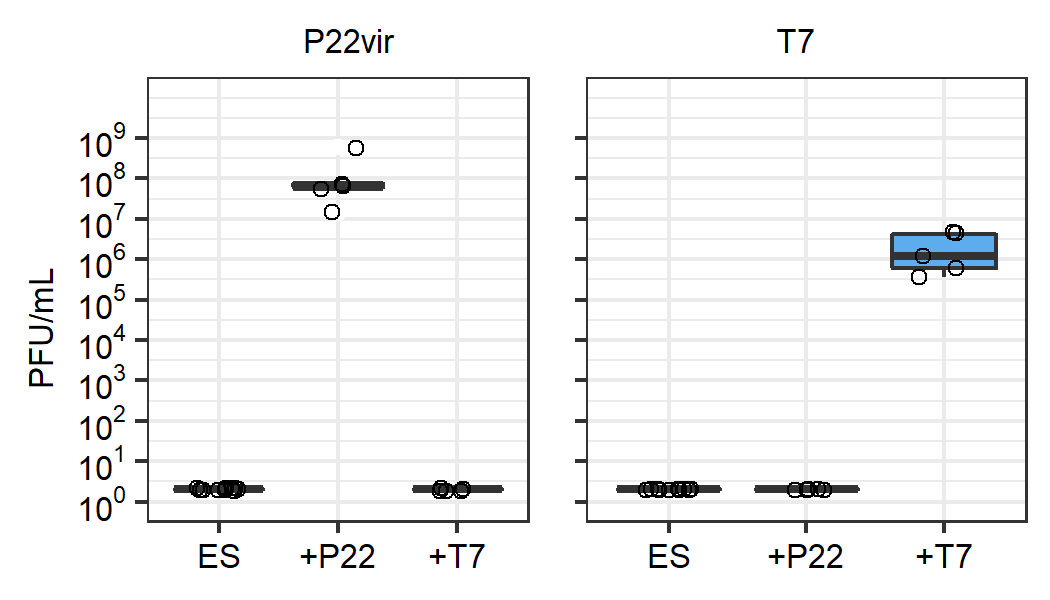


**Supplementary Figure 4. Phage PFU measurements from wet-lab co-culture experiments**. Phage titers of **A)** P22*vir* and **B)** T7 were measured for no phage controls (ES, n=10), co-culture with P22*vir* (+P22, n = 5), and co-cultures with T7 (+T7, n = 5).

**
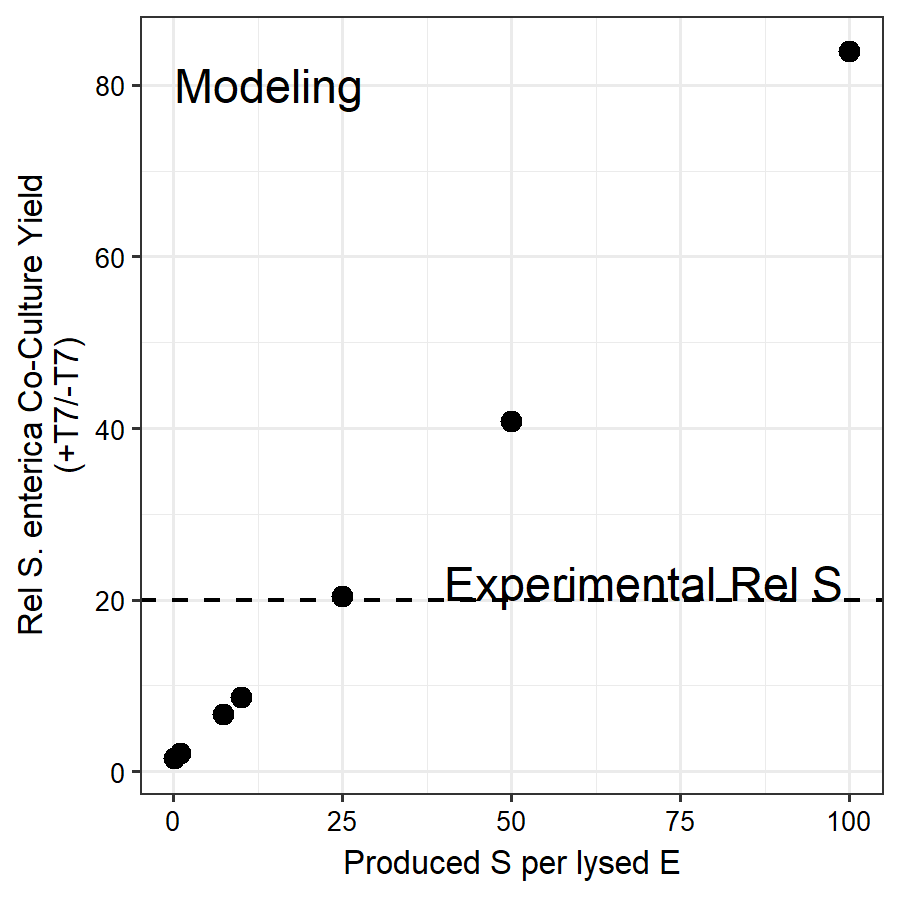
**

**Supplementary Figure 5. Mathematically modeling a range of cellular debris conversion rates shows quantitative differences in non-host final yields.** In mathematical models, conversion rates were simulated by including a scaling variable for the production of cellular debris parameter by *E. coli*. Simulating experimental relative S yields required production of ~ 25 *S. enterica* per lysed *E. coli*.

**Supplementary Table 1. Resource-explicit mathematical model parameters.**

| Parameter | Description | Value | Units | Reference |
| --- | --- | --- | --- | --- |
| μ_E_ | E, Es, Er growth rate (hr^-1^) | 0.550 (lcts) | h^-1^ | This study |
| μ_S_ | S, Ss, Sr growth rate (hr^-1^) | 0.156 (ac) | h^-1^ | This study |
| p_ac_ | Es & Er acetate production (base) | 4 x 10^-13^  6 x 10^-13^ | g/cell | [3] (Adjusted) |
| p_met_ | S methionine production | 1 x 10^-12^ | g/cell | [3] (Adjusted) |
| p_cd_ | E production of cellular debris | 2 x 10^-12^ | g/cell | This study |
| c_ac_ | S acetate consumption | 3 x 10^-12^ | g/cell | [3] (Adjusted) |
| c_cd_ | S cellular debris consumption | 2 x 10^-12^ | g/cell | This study |
| c_lcts_ | Es & Er lactose consumption | 2 x 10^-12^ | g/cell | [3] (Adjusted) |
| c_met_ | Es & Er methionine consumption | 5 x 10^-14^ | g/cell | [3] (Adjusted) |
| β | Burst size | 100 | phage progeny/infected cell | [4] (Adjusted)^a^ |
| γ | Adsorption rate (successful infection per encounter) | 10^-9^ | min^-1^ | [4] (Adjusted)^a^ |
| k_ac_ | Michaelis-Menton acetate saturation | 3 x 10^-7^ | g/200μl | [3] (Adjusted) |
| k_cd_ | Michaelis-Menton cellular debris saturation | 3 x 10^-7^ | g/200μl | This study |
| k_lcts_ | Michaelis-Menton lactose saturation | 7 x 10^-7^ | g/200μl | [3] (Adjusted) |
| k_met_ | Michaelis-Menton methionine saturation | 3 x 10^-7^ | g/200μl | [3] (Adjusted) |

^a^ De Paepe & Taddei (2006) [4] used *E. coli* strain MG1655 at 37°C in LB to measure parameters. We decreased our model burst rate (β) and adsorption rate (γ) parameters to accommodate slower growth in 30°C growth in lactose minimal media.

**Supplementary Table 2. Measured starting densities and MOIs.**

| Experiment # | Community Type | E (cells/well) | S (cells/well) | Phage (Type) (PFU/well) | MOI |
| --- | --- | --- | --- | --- | --- |
| 1 | ES | 2.25x10^5^ | 1.77x10^5^ | NA | NA |
| 1 | ES + T7 | 2.25x10^5^ | 1.77x10^5^ | 1.6x10^3^ (T7) | 0.007 |
| 2 | ES | 1.1x10^5^ | 1.16x10^4^ | NA | NA |
| 2 | ES + P22*vir* | 1.1x10^5^ | 1.16x10^4^ | 2.1x10^2^ (P22*vir*) | 0.018 |

| Supplementary Table 3. Phage communities cross-streaked against evolved isolates. | | | | | | | | |
| --- | --- | --- | --- | --- | --- | --- | --- | --- |
| Bacterial Isolates | **Host Species** | **Comm. Rep #^a^** | **Community Treatment** | **Phage Lysate Community Replicate #^b^** | | | | |
|  |  |  |  | **1** | **2** | **3** | **4** | **5** |
| Anc E | *E. coli* | NA | NA | S | Growth | S | Growth | S |
| 1a | *E. coli* | 1 | ES + T7 | PR (m) | R (m) | R (m) | R (m) | R (m) |
| 1b | *E. coli* | 1 | ES + T7 | PR (m) | R (m) | R (m) | R (m) | R (m) |
| 1c | *E. coli* | 1 | ES + T7 | PR (m) | R (m) | R (m) | R (m) | R (m) |
| 3a | *E. coli* | 3 | ES + T7 | PR (m) | R (m) | R (m) | R (m) | R (m) |
| 3b | *E. coli* | 3 | ES + T7 | PR (m) | R (m) | R (m) | R (m) | R (m) |
| 3c | *E. coli* | 3 | ES + T7 | PR (m) | R (m) | R (m) | R (m) | R (m) |
| 5a | *E. coli* | 5 | ES + T7 | PR (m) | R (m) | R (m) | R (m) | R (m) |
| 5b | *E. coli* | 5 | ES + T7 | PR (m) | R (m) | R (m) | R (m) | R (m) |
| 5c | *E. coli* | 5 | ES + T7 | PR (m) | R (m) | R (m) | R (m) | R (m) |
| Anc S | *S. enterica* | NA | NA | S | S | S | S | S |
| 1a | *S. enterica* | 1 | ES + P22*vir* | R (nm) | R (nm) | R (nm) | R (nm) | R (nm) |
| 1b | *S. enterica* | 1 | ES + P22*vir* | No Growth | No Growth | No Growth | No Growth | No Growth |
| 1c | *S. enterica* | 1 | ES + P22*vir* | R (nm) | R (nm) | R (nm) | R (nm) | R (nm) |
| 2a | *S. enterica* | 2 | ES + P22*vir* | R (nm) | R (nm) | R (nm) | R (nm) | R (nm) |
| 2c | *S. enterica* | 2 | ES + P22*vir* | R (nm) | R (nm) | R (nm) | R (nm) | R (nm) |
| 2b | *S. enterica* | 2 | ES + P22*vir* | R (nm) | R (nm) | R (nm) | R (nm) | R (nm) |
| 3a | *S. enterica* | 3 | ES + P22*vir* | R (nm) | R (nm) | R (nm) | R (nm) | R (nm) |
| 3b | *S. enterica* | 3 | ES + P22*vir* | R (nm) | R (nm) | R (nm) | R (nm) | R (nm) |
| 3c | *S. enterica* | 3 | ES + P22*vir* | R (nm) | R (nm) | R (nm) | R (nm) | R (nm) |
| 4a | *S. enterica* | 4 | ES + P22*vir* | R (nm) | R (nm) | R (nm) | R (nm) | R (nm) |
| 4b | *S. enterica* | 4 | ES + P22*vir* | R (nm) | R (nm) | R (nm) | R (nm) | R (nm) |
| 4c | *S. enterica* | 4 | ES + P22*vir* | R (nm) | R (nm) | R (nm) | R (nm) | R (nm) |
| 5a | *S. enterica* | 5 | ES + P22*vir* | R (nm) | R (nm) | R (nm) | R (nm) | R (nm) |
| 5b | *S. enterica* | 5 | ES + P22*vir* | R (nm) | R (nm) | R (nm) | R (nm) | R (nm) |
| 5c | *S. enterica* | 5 | ES + P22*vir* | R (nm) | R (nm) | R (nm) | R (nm) | R (nm) |
| ^a^ *E. coli* in ES + T7 communities #2 and #4 went extinct. | | | | | | | | |
| ^b^ S = Sensitive, PR = Partial Resistant, R = Resistant, (nm) = non-mucoid, (m) = mucoid | | | | | | | | |

| Supplementary Table 4. Cellular debris conversions – cells produced per lysed cell equivalents. | | | | |
| --- | --- | --- | --- | --- |
| Bacterial Cellular Debris Provided | **# Cell-equivalents of cellular debris provided** | **Bacterial Species Produced** | **# Cells Produced** | **# Cells Produced Per Cell Lysed** |
| *E. coli* | 1.8x10^7^ | *S. enterica* | 1.32x10^8^ | 7.33 |
| *E. coli* | 1.8x10^7^ | *E. coli* | 6.03x10^5^ | 0.03 |
| *S. enterica* | 4.1x10^7^ | *S. enterica* | 2.56x10^6^ | 0.06 |
| *S. enterica* | 4.1x10^7^ | *E. coli* | 1.92x10^6^ | 0.05 |
